# An Amazingly Simple, Fast and Green Synthesis Route to Polyaniline Nanofibers for Efficient Energy Storage

**DOI:** 10.3390/polym12102212

**Published:** 2020-09-27

**Authors:** Sami ur Rahman, Philipp Röse, Anwar ul Haq Ali Shah, Ulrike Krewer, Salma Bilal

**Affiliations:** 1 National Centre of Excellence in Physical Chemistry 1, University of Peshawar, Peshawar 25120, Pakistan; samiurrahman364@yahoo.com; 2 Karlsruhe Institute of Technology (KIT), Institute for Applied Materials—Materials for Electrical and Electronic Engineering (IAM), 76131 Karlsruhe, Germany; philipp.roese@kit.edu; 3 Institute of Chemical Science, University of Peshawar, Peshawar 25120, Pakistan; anwarulhaqalishah@uop.edu.pk

**Keywords:** polyaniline, supercapacitors, nanofibers, sodium phytate, conductive polymer

## Abstract

The major drawbacks of the conventional methods for preparing polyaniline (PANI) are the large consumptions of toxic chemicals and long process durations. This paper presents a remarkably simple and green route for the chemical oxidative synthesis of PANI nanofibers, utilizing sodium phytate as a novel and environmentally friendly plant derived dopant. The process shows a remarkable reduction in the synthesis time and usage of toxic chemicals with good dispersibility and exceedingly high conductivity up to 10 S cm^−1^ of the resulting PANI at the same time. A detailed characterization of the PANI samples has been made showing excellent relationships between their structure and properties. Particularly, the electrochemical properties of the synthesized PANI as electrode material for supercapacitors were analyzed. The PANI sample, synthesized at pre-optimized conditions, exhibited impressive supercapacitor performance having a high specific capacitance (C_sp_) (832.5 Fg^−1^ and 528 Fg^−1^ at 1 Ag^−1^ and 40 Ag^−1^, respectively) as calculated from galvanostatic charge/discharge (GCD) curves. A good rate capability with a capacitance retention of 67.6% of its initial value was observed. The quite low solution resistance (R_s_) value of 281.0 × 10^−3^ Ohm and charge transfer resistance value (R_ct_) of 7.44 Ohm represents the excellence of the material. Further, a retention of 95.3% in coulombic efficiency after 1000 charge discharge cycles, without showing any significant degradation of the material, was also exhibited.

## 1. Introduction

Presently there are deep concerns over the environmental consequences of high energy demands owing to the rapid increase in the usage and availability of energy and resource intensive technologies. The high consumption of non-renewable sources of energy with the related greenhouse effects prompted huge interest in the use of renewable energy sources and development of clean and competent energy devices [1].

From these various energy storage devices, supercapacitors (SCs) in particular have gained incredible research attention due to their low cost, fast charging and discharging rate, excellent power density and long cycle life [2]. Their capability to deliver stored energy with high density makes them an outstanding class of energy storage devices. Typically, their electric charge is stored at the electrode/electrolyte interface [3]. The amount of stored energy in SCs originates from non-faradic current and pseudo-capacitance. The charging of the double layer is responsible for the origination of energy from non-faradic current, while energy of the pseudo-capacitance comes from faradic current which arises by the oxidation/reduction processes taking place at the electrode surface modified with active material [4,5,6]. Due to their above-mentioned properties pseudo-capacitive electrode materials occupy a substantial role in the performance of the energy storage and conversion devices. In the quest for even more efficient electrode materials with superior performance, material scientists are consistently looking for candidates.

Polyaniline (PANI), a promising member of the class of intrinsically conducting polymers (ICPs), is at the forefront of global research [7]. It has the advantages of excellent physicochemical properties and the capability to conduct both electronic and ionic charges [8]. The conductivity of PANI results from a process known as “doping”. In this procedure the polymer is treated with agents called dopants. These dopants have the ability to add or remove electrons from the backbone of the treated polymer [9]. The phenomenon in PANI makes it potent for insulator to conductor transition. Agitation of the dopant amount with the polymeric back bone changes the electrical properties of the polymers [10].

PANI’s are widely used in energy storage and conversion devices due to their high energy storage capability through multiple redox transitions, high conductivity, and their flexibility in synthesis [11]. The performances strongly depend on their structure, particularly, their specific surface area. Nanosized particles with large specific surface area are known to be the first choice when high capacitances are desired [12]. Wang and co-workers [13], showed that electrochemically polymerized PANI with unique nanowire structure as active material for SCs exhibited a high capacitance of 950 Fg^−1^. Moreover, Sivakkmar et al. [14], synthesized PANI nanofibers through interfacial polymerization and achieved high capacitances up to 554 Fg^−1^. Fahim et al. [15] fabricated PANI as electrode material in symmetric SC exhibiting a high capacitance of 712 Fg^−1^ at 0.5 Ag^−1^. One major advantage is that PANI alone can be used in fabricating an electrode without adding other additives or binders [7].

The reported methods to prepare nanostructures PANI, including electrochemical oxidative polymerization [16,17], interfacial polymerization [15,18], radical polymerization [19], seeding polymerization [20], microwave assisted polymerization [20,21] and chemical oxidative polymerization are the most common [13,22,23]. Unfortunately, low processability and dispersibility due to the stiffness of backbone and H-bonding interactions between the adjacent chains remained the major problems associated with the PANI based materials that bound their applications in practical fields. In the context of energy storage, PANI suffers from fading of capacitance at high current density or long-term cycling. So far, the literature, available on PANI, indicates that a certain desired property of PANI is accomplished by the sacrifice of other promising properties, e.g., an improvement in solubility most often results in a decrease in conductivity.

Hence, there is a need to look for alternate synthesis methodologies where PANI can retain all its valuable characteristic properties. Further, an inexpensive and less environmentally hazardous approach (by minimizing the use of toxic chemicals) is worthwhile. It can facilitate its production without any threat to the environment and certainly increase its demand for application in various electrochemical technologies.

In the present work, we have synthesized highly conductive PANI nanofibers as electrode material for SCs by a remarkably simple, scalable, cost-effective, and rapid process by using plant derived sodium phytate as a novel dopant. Sodium phytate is an abundant plant derived pollution free compound having a good solubility in water. It acts both as dopant and cross linker and form phosphorylated PANI with interconnected net like structure. The synthesis protocol covers multiple aspects of synthesis and energy related issues as the polymerization time and use of toxic chemicals is reduced by using water as a reaction medium instead of organic solvents in comparison to the previously reported methods (see Supplementary Materials, Table S1). The material is highly conductive and dispersible in a number of solvents and was checked as electrode material for SCs by electrochemical characterization, exhibiting excellent energy storage properties at optimized conditions. Therefore, this simple and scalable synthesis procedure of the synthesized materials and its excellent electrochemical performance provides a gateway to cost effective SCs electrodes.

## 2. Experimental Section

### 2.1. Materials and Methods

The PANI samples were synthesized from commercially available Stock: aniline (C_6_H_5_NH_2_), sodium phytate (C_6_H_17_NaO_24_P_6_), ammonium persulphate ((NH_4_)_2_S_2_O_8,_ dimethylformamide (C_3_H_7_NO) were purchased from Sigma Aldrich (St. Louis, MI, USA) and sulfuric acid (H_2_SO_4_) was provided by Scharlau (Barcelona, CAT, Spain). Aniline was freshly distilled twice to remove any types of impurities. After distillation, the aniline was kept in a refrigerator for further use. Gold foil (99.99%, 0.127 mm thickness) was purchased from ChemPur (Karlsruhe, BW, Germany). The rest of the chemicals was used as received. Deionized water was utilized for sample synthesis and washing purposes.

### 2.2. General Procedure for the Synthesis of Sodium Phytate Doped PANI

Solutions with 0.005 M (0.5%), 0.01 M (1%), 0.03 M (3%), 0.05 M (5%), 0.07 M (7%) and 0.10 M (10%) sodium phytate were prepared in H_2_O at room temperature (solution A). Then 2.5 mL from the respective sodium phytate solution was mixed with 497 μL (5.50 mmol) aniline in 5 mL H_2_O. A second solution containing ammonium persulfate (1.0 mM in H_2_O) was prepared (solution B). These solutions were kept in the refrigerator for 15 min. After that polymerization was performed by adding 1.0 mL from solution A to 0.5 mL of solution B in an Eppendorf tube (solution C), followed by thorough mixing (see Supplementary Materials, Figure S1). After 5 min, the color of the mixture turned dark green indicating the formation of PANI and the reaction was finished after 10 min. The mixture was filtered, washed with acetone and water, and dried under vacuum. The respective PANI samples were labeled according their amount of dopant with P-0.5PA for the lowest to P-10PA with highest dopant concentration.

### 2.3. Fabrication of PANI on Gold Electrode

For gold electrodes the surface was polished with diamond paste and rinsed with water and isopropanol and dried at 100 °C for 24 h. 10 mg of PANI-sample (S1 (P-0.5PA), S2 (P-1PA), S3 (P-3PA), S4 (P-5PA), S5 (P 7PA), S6 (P-10PA)) was suspended in 5 mL DMF and stirred thoroughly at room temperature, then placed in an ultrasonic sound bath for 30 min. The respective gold sheet was coated with PANI-DMF suspension. Then the solvent was led to evaporate slowly at 25 °C for 12 h resulting in a thin and even layer of dry PANI on the gold surface. The coated PANI-gold electrode was dried under vacuum at 80 °C for 24 h to remove residual solvent. After drying the active PANI material on the gold electrode surface was 2 mg/cm^2^ for each sample analysis. Before electrochemical measurements were done, there was a 45 min resting time to get a constant temperature of 25 °C.

### 2.4. Characterization

#### 2.4.1. Structural and Morphological Characterization

Surface morphology, elemental composition, and mapping of the synthesized PANI samples were done by Helios G4 CX FEI Deutschland GmbH. Nitrogen absorption-desorption isotherms were measured using Brunauer–Emmett–Teller (BET) method on a surface area analyzer from Quanta Chrome Instrument (Version 11.04, Boynton Beach, FL, USA). The pore size distribution was obtained from the adsorption branches by using the Barret–Joyner–Halenda (BJH) method. A Shimadzu Affinity-1S FT-IR spectrometer was used for molecular structure and functional group identification. UV-Vis experiments were performed on a LAMBDA 1050 from Perkin Elmer (Waltham, MA, USA). The conductivity was determined in the form of pellets by using a four probe conductometer (Jandel RM 3000, Jandel Engineering Ltd., Linslade, Beds., UK) equipped with a potentiostat. The pellets (diameter: 13 mm and thickness: 5 mm) were made by hydraulic press using a pressure of 15 tons.

#### 2.4.2. Electrochemical Characterizations

Electrochemical performance of the material was studied by using ZRA/Potentiostat/Galvanostat Reference 3000. The catalytic activity and redox processes of the synthesized PANI were investigated by using cyclic voltammetry (CV, Gamry Instruments, Warminster, PA, USA) measurement at different scan rates within the potential ranging from −0.2 V to 0.8 V. The experiment was carried out in a three-electrodes system using 1M H_2_SO_4_ electrolyte, consisting gold sheet (working electrode on which surface the material was coated), Ag/AgCl (reference electrode) and gold coil (counter electrode). For coating the electrode, the appropriate amount of powder sample was dissolved in DMF and then the active material was drop coated on the working electrode. From CVs curves, the specific capacitance (C_sp_) of various PANI salts were calculated by using the equation [24]:

(1) $$C_{sp}=\frac{I}{mv}$$

where ‘*I*’ signifies the current, ‘*m*’ depicts the mass of the active material and ‘*v*’ represents the scan rate. The active material loading was 2 mg cm^−2^ (dry weight).

The galvanostatic charge/discharge (GCD) measurement of the most optimized sample P-5PA was done in a potential ranging from −0.2 to 0.8 V at various current densities (1, 3, 5, 10, 15, 20, 30 and 40 Ag^−1^), respectively. The experiment was performed in the same manner as discussed in CV, using three electrode systems. From GCD curves, the specific capacitances (*C_sp_*) of the PANI salt (P-5PA) was calculated by using the equation [25]:

(2) $$C_{sp}=\frac{I\cdot \Delta t}{\Delta v\cdot m}$$

where, *C_sp_* designates specific capacitance (Fg^−1^), Δ*t* is the time period of the discharging process in seconds (s), *I* refers to the current (*A*), Δ*V* tells the potential window in volts (*V*), *m* denotes the mass of the electroactive material in gram. Electrochemical Impedance Spectroscopy (EIS, Gamry Instruments, Warminster, PA, USA) was also used to characterize electroactive materials for charge transfer resistance. During the EIS measurement the frequency range was between 0.1 Hz and 1.0 MHz at a potential range of 0.4–0.8 V.

## 3. Results and Discussion

### 3.1. Polymerization of Aniline

**Scheme 1:**
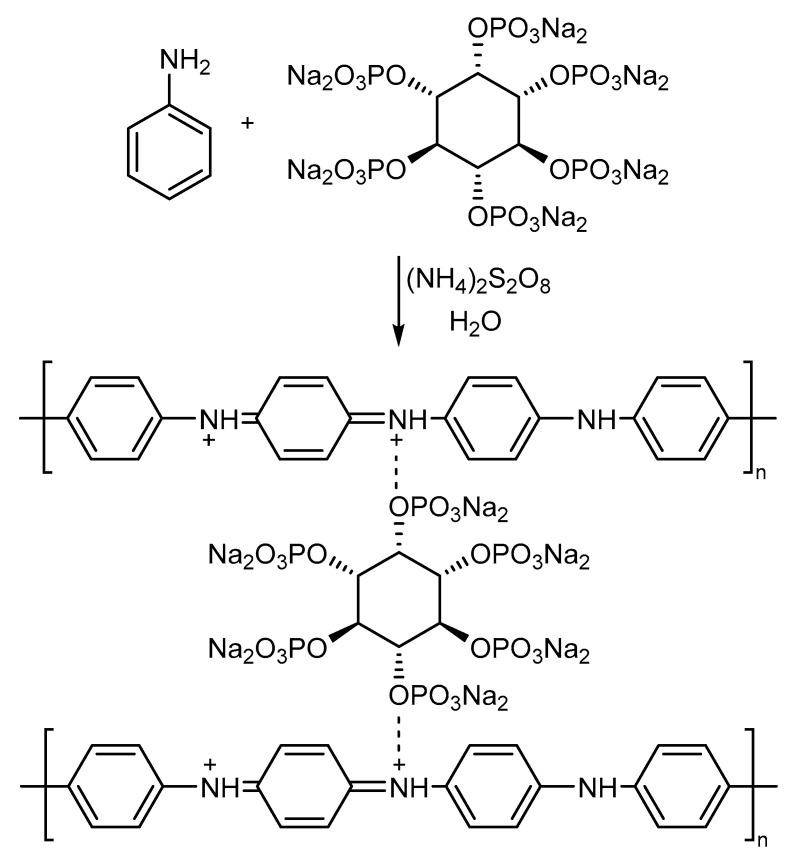
Synthesis mechanism of sodium phytate doped polyaniline (PANI).

Chemical oxidative polymerization is a traditional technique used for the preparation of PANI in bulk with a large consumption of chemicals and a long process time [26,27]. The polymerization is usually performed by the slow addition of reactants one after another under vigorous stirring. The obtained product is often highly agglomerated and irregularly shaped because of heterogeneous nucleation. The key to obtain PANI nanofibers is to overcome the heterogeneous nucleation [28,29]. In the present study, we rapidly mixed aniline and sodium phytate solution with the oxidant at room temperature (Scheme 1). This allowed an even distribution of monomer and oxidant molecules before polymerization. When the polymerization started, the oxidant rapidly polymerized the monomers in less than 15 min and induced the formation of nanofibers by overcoming the heterogeneous nucleation. This led to the consumption of all the oxidant, dopant, and monomer molecules to form the respective PANI in remarkably high yield (98%) in the optimized sample P-5PA by a reproducible, inexpensive, and scalable method. To the best of our knowledge there is no report on the synthesis of PANI that is as green, efficient, rapid, and simple.

The PANI samples were dispersible in DMSO, DMF, NMP, THF, ethanol etc., which permitted the use of facile and cost-effective techniques to process this synthesized nanostructure material into a variety of utilizable forms like films and composites. This makes the protocol to a time- and cost effective, less toxic, and efficient strategy for improvement in PANI processability compared to prior work (see Supplementary Materials, Table S1) [30,31].

### 3.2. Structural and Morphology Characterization

**Figure 1:**
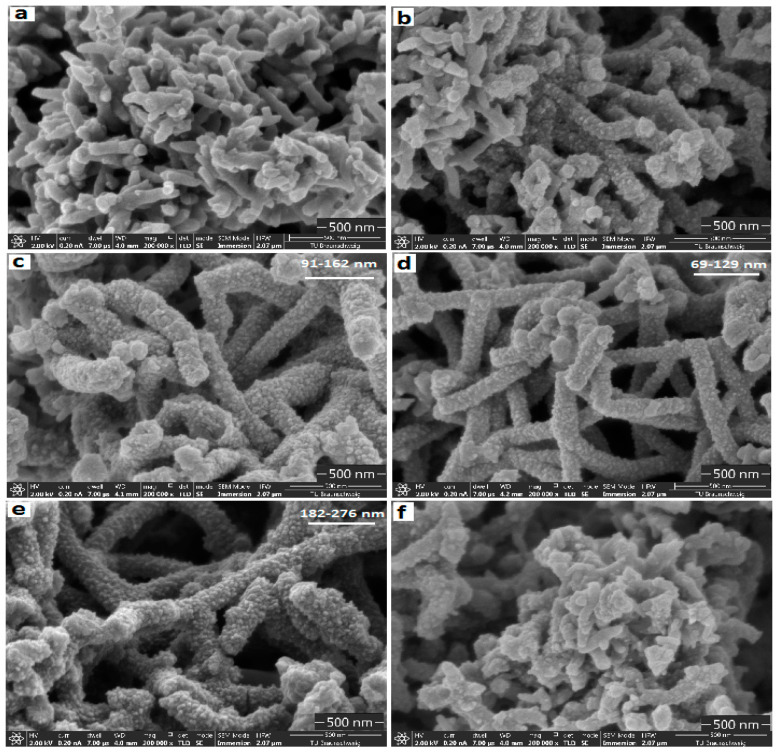
SEM pictures of the surface of PANI: (**a**) P-0.5PA, (**b**) P-1PA, (**c**) P-3PA, (**d**) P-5PA, (**e**) P-7PA, (**f**) P-10PA.

Figure 1 shows the different morphologies of the PANI with variant sodium phytate dopant amounts. For P-0.5PA and P-1PA (Figure 1a,b) irregular agglomerated short fibrous growth patterns were found. With increasing amount of dopant, e.g., 3% for P-3PA (Figure 1c), the morphology changed to dense interconnected, branched, and twisted fibers with a diameter of 91 to 162 nm. While this structure was already more fiber-like, it was still not feasible for good energy storage applications as the presence of branched structures and twists hinder the diffusion of electrolyte into the polymer matrix [32,33].

In contrast, for P-5PA long and uniform fiber interconnected structures with rough and porous morphology were observed (Figure 1d). The fibers had diameters of 69 to 129 nm and their structure revealed no random agglomerates as within the other samples. Further increase in dopant amount to 7% (P-7PA) resulted in the formation of thicker and shortened fibers with the appearance of random aggregates, with a diameter range of 182 to 276 nm (Figure 1e). Breakage of long fibers into small pieces was clearly visible with increase in diameter. This in turn was expected to reduce the electrical properties of the material [34]. Those short fibers completely agglomerate when the dopant amount is further increased to 10% (P-10PA) (Figure 1f).

The differences in morphology relate directly to the surface area value, pore size distribution range and volume of the pores. The surface area of the P-0.5PA, P-5PA and P-10PA samples were calculated by measuring nitrogen absorption and desorption isotherms using Brunauer–Emmett–Teller (BET) analysis (see Supplementary Materials, Figures S2–S4). The P-5PA exhibited a significantly higher surface area value (230.5 m^2^g^−1^) than the P-0.5PA (200.4 m^2^g^−1^) and the P-10PA (101.5 m^2^g^−1^). Furthermore, the pore size distributions and volumes were measured by the Barrett–Joyner–Halenda (BJH) method. The pore size distribution of the samples were similar, ranging from 15.0–19.5 nm while the volume followed the same trend as with the surface area with 0.032 cm^3^g^−1^ for P-0.5PA, 0.046 cm^3^g^−1^ for P-5PA and 0.011 cm^3^g^−1^ for P-10PA, respectively.

The results revealed that the reaction parameters of P-5PA with 5% sodium phytate were most desirable to achieve fibrous and porous nanostructure networks with distinct connectivity, particle size distribution and pore volume. As well-known, such interconnected PANI nanofiber structures can be more beneficial for electrical and capacitive properties than wires and particles when used as electrode material for supercapacitors. The large open pore channels within the structure [35], as well as a large fibrous and rough surface area favor ion transport and charge transfer reactions, which are beneficial for good electrocatalytic properties [28,36,37].

For an even more detailed insight into the elemental composition of the sodium phytate doped PANI samples EDX analysis was employed (see Supplementary Materials, Figures S5–S7)

The spectra revealed that all PANI samples contain C, O, N, Na and P. EDX-mapping also established a homogeneous distribution of the elements. This evidently proved the successful synthesis of PANI with effective integration of dopant into the polymer backbone. Of particular importance were the Na and P contents since they correlate directly with the dopant amount. With increasing dopant/aniline ratios in synthesis a steady increase in Na and P contents up to the P-5PA sample could be observed (Table 1). Further increase in ratios resulted in a significant decrease in dopant assimilation during synthesis. Especially the high content in Phosphor of the sample P-5PA is worthwhile, because it reduces the agglomeration of the polymer chains and barrier height, creates a deep interaction for intra- and inter-molecular charge delocalization and is expected to enhance the electrical conductivity [38].

**Figure 2:**
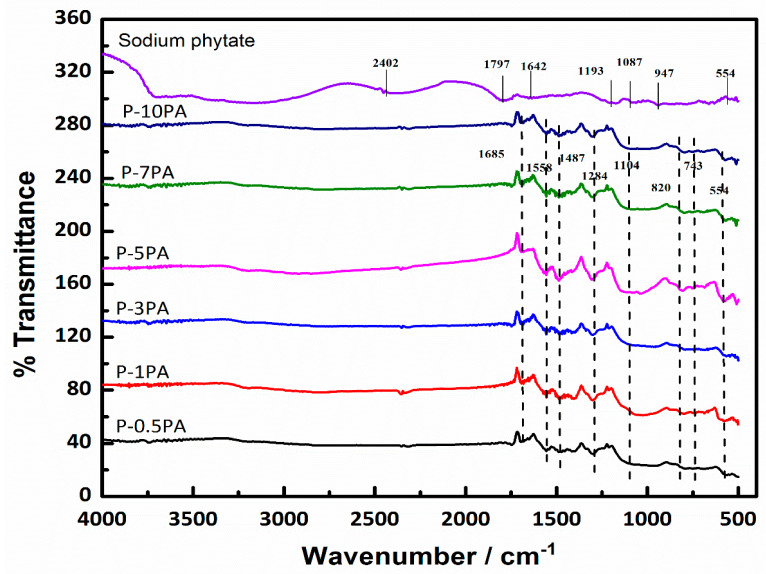
FT-IR spectra of sodium phytate and P-0.5PA to P-10PA.

Figure 2 depicts the FT-IR spectra of sodium phytate and the synthesized PANI samples. The FT-IR spectrum of sodium phytate displays basic absorption peaks at 1193 and 1087 cm^−1^ that can be assigned to the stretching vibration of P=O and P-O–C, respectively. The peak at 554 cm^−1^ corresponds to O-Na interaction, while the one at 2402 cm^−1^ is due to O–P=O vibrations. The peaks of –OH group of present water molecules in the sodium phytate salt appear at 1642 and 1797 cm^−1^ [39].

In the FT-IR spectra of the PANI samples, the characteristic peaks at 1685 and 1558 cm^−1^ are attributed to the vibrations of C═C in the quinoid and benzenoid rings, respectively. The typical peak at 1487 cm^−1^ is assigned to C–N stretching vibrations in the neighborhood of the quinoid ring [40]. The absorption at 1284 cm^−1^ is attributed to the C–N bonds associated with the benzenoid ring. The absorption at 1104 cm^−1^ is due to the C═N bond associated with the quinoid ring. Those peaks are characteristic for PANI in its emeraldine form where the doping primarily occurs at the quinoid ring segments. Additionally, the broadened band at 1104 cm^−1^ indicates an electron delocalization in the PANI backbone, which guarantees a high conductivity [41,42,43]. Furthermore, the peaks at 820 and 554 cm^−1^ are associated with P–O–C and O-Na stretching vibrations of stray free phosphonates of the phytic acid sodium salt [41,44].

**Figure 3:**
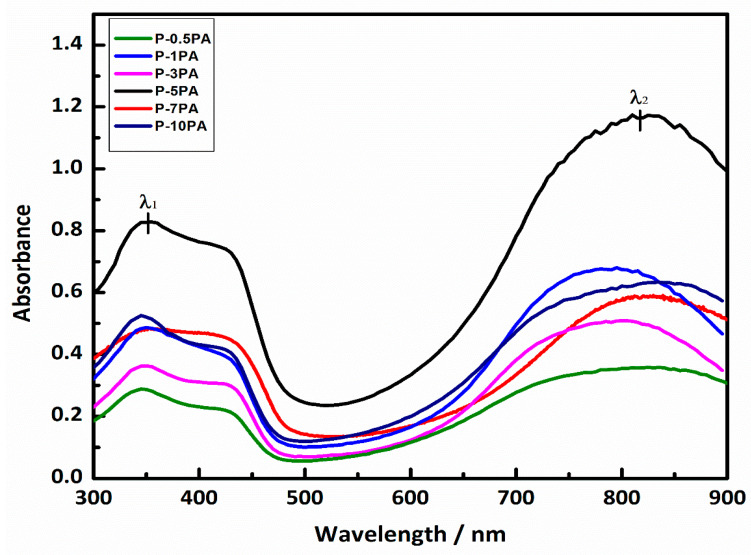
UV-vis spectra of the sodium phytate doped PANI samples.

Further validation of the PANI structure was employed by UV-vis analysis (Figure 3). The spectra revealed three major absorbance peaks. The first peak at 345 nm (λ_1_) was attributed to the π-π* transition by excitation of nitrogen of the benzenoid rings. The other two peaks at 424 (λ_2_) nm and 787 nm (λ_3_) belong to polaron-π* transition and π-polaron transition, respectively, indicating that the samples are in the conductive emeraldine form [45]. The transition band at 787 nm is strongly broadened, which is certainly caused by interband charge transfer from benzenoid to quinoid rings of conjugated PANI. It is well-known that λ_3_ exhibits a significant red shift with increase in dopant concentration, consequently, the stronger the absorbance of this band the more dopant is incorporated and the larger is the conjugation length and ordered structure of the PANI. This can be clearly seen for P-1PA to P-5PA. As in accordance with SEM and EDX data, P-7PA and P-10PA revealed blue-shifts compared to P-5PA, which relate to less amounts of incorporated dopant and shorter chain length.

### 3.3. Electrochemical Properties

To confirm that the PANI samples have the potential as electrode material for supercapacitors, the electrical and electrochemical properties were investigated by conductivity measurements, cyclic voltammetry (CV), galvanostatic charge/discharge (GCD), and electrochemical impedance spectroscopy (EIS).

The electrical (DC) conductivity of the PANI samples was determined from conductance measurements multiplied by the geometrical factor. Before the measurement, the respective powders of the PANI samples were pressed into small pallets (13 mm Ø, 5 mm thickness) using a hydraulic press with 15 tons of load. The four-probe method is ideal for those kinds of samples, because it eliminates the influence of leads resistance or contact resistance, providing a better accuracy for the measuring conductance [46]. The conductivity values at 25 °C of the PANI samples are shown in Table 2.

The results illustrate an increase in conductivity with increasing amount of dopant content up to P-5PA with remarkable 10 S cm^−1^. Then, for P-7PA and P-10PA the conductivity decreases significantly. The differences in conductivity are in accordance with the SEM, BET and EDX results as SEM results revealed long and uniform fiber interconnected structures with rough and porous morphology for P-5PA. The same sample exhibited large surface area and small pore volume during BET analysis while highly assimilated dopant content of Na and P was also observed for sample P-5PA suggesting that uniform interconnected fibrous and porous nature with large surface area and high dopant contents favors high conductivity. Moreover, the conductivities of the presented sodium phytate doped PANI samples are a magnitude higher than those using phytic acid solution (0.11 S cm^−1^ [37], 0.28 S cm^−1^) [38], or phytic acid/hydrochloric acid mixtures (0.48–0.61 S cm^−1^) [47], during synthesis as well as with other dopants [48,49,50].

#### 3.3.1. Cyclic Voltammetry (CV)

**Figure 4:**
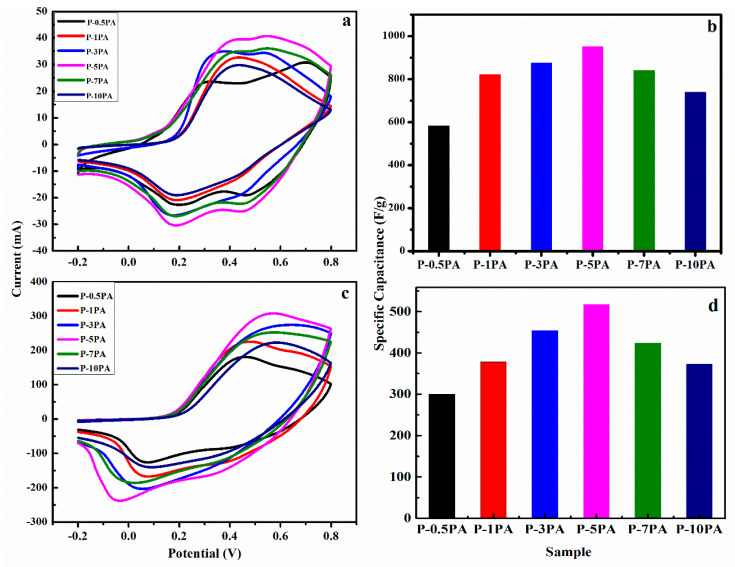
Cyclic voltammetry (CV) curves of various PANI salts in 1 M H_2_SO_4_ solution (**a**) at scan rate of 20 mVs^−1^; (**b**) histogram showing C_sp_ values calculated from CV curves at scan rate of 20 mVs^−1^; (**c**) at scan rate of 300 mVs^−1^; (**d**) histogram showing C_sp_ values calculated from CV curves at scan rate of 300 mVs^−1^.

To investigate the dopant influence on electrocatalytic efficiency and capacitive energy storage behaviors of the PANI salts, the electrochemical properties of the synthesized samples (P-0.5PA, P-1PA, P-3PA, P-5PA, P-7PA and P-10PA) as electrode in SCs were tested by CV (Figure 4). The curves were also used to calculate the specific capacitances of the electrode materials at different scan rates by using equation [24]. The compared CV profile of the PANI electrodes (P-0.5PA, P-1PA, P-3PA, P-5PA, P-7PA and P-10PA) were recorded at a lower scan rate (20 mVs^−1^) and are depicted in Figure 4a. All the PANI salts illustrate a quasi-rectangular shaped CV curve with two pairs of redox peaks that are attributable to the conversion of leucomeraldine to emeraldine form and further to pernigraniline [51]. The shape is typical for PANI and consists of a combination of double layer capacitance and pseudo capacitance with its two redox pairs [52]. It can be seen that among different samples of PANI salts, P-5PA exhibits a significantly high current, suggesting its strong interaction with the dopant. Such interaction results in high porosity and rough surface, providing larger electrode/electrolyte interface area for more redox reactions [53,54] and provides fast rate of ionic transport and high capacitance [51]. The specific capacitances calculated from the curves (at low scan rate 20 mVs^−1^) are presented in Table 3 and histogram (Figure 4b). The obtained results show that P-5PA exhibits a higher value of C_sp_ (950.0 ± 1.09 Fg^−1^) in comparison with P-0.5PA (582.5 ± 2.06Fg^−1^), P-1PA (819.8 ± 1.81 Fg^−1^), P-3PA (875.0 ± 1.55 Fg^−1^), P-7PA (840.0 ± 1.95 Fg^−1^) and P-10PA (739.0 ±2.24 Fg^−1^).

The samples were further tested by subjecting them to higher scan rate of 300 mVs^−1^ and the obtained curves are displayed in Figure 4c. The specific capacitances are given in table (Table 3) and histogram (Figure 4d). It can be observed from the CV curves that the rectangular shape of all the samples are maintained even at higher scan rate which describes its excellent electrochemical performance. Shifting of anodic peaks towards positive potential is obvious because at higher scan rate, there is an enhancement of ohmic resistance which makes diffusion of ions into the inner surface of electrode material difficult and is expected to peak shifting [55,56]. Additionally, lower specific capacitance is observed for all the samples at higher scan rate. P-5PA shows the highest specific capacitance of 517.5 ±1.52 Fg^−1^ relative to all other samples i.e., P-0.5PA (300.5 ± 2.38 Fg^−1^), P-1PA (378.7 ± 2.10 Fg^−1^) P-3PA (454.2 ± 1.62 Fg^−1^), P-7PA (424.2 ± 1.96 Fg^−1^) and P-10PA (372.7 ± 2.05 Fg^−1^).

From the tabulated values, it can be observed that the capacitance is inversely related to the scan rate as all the samples show high capacitance values at low scan rate than at a high scan rate. It is thought that low scan rate can permit electrolyte to easily enter the pores of the active material and interact with the electrode’s inner surface and lead to large storage of charge on the electrode surface, hence provide high capacitance. Whereas at higher scan rate, an ineffective interaction occurs between the electrode and electrolyte due to diffusion limitation, which results in less storage of charge at the surface of electrode, hence a low capacitance is observed [54].

The above observations determine that P-5PA exhibits the highest specific capacitance both at lower and higher scan rates. The superior capacitive performance attained by our synthesized material endorses the distinctive advantages of the rough, porous interconnected nanostructures and sodium phytate crosslinked PANI material.

**Figure 5:**
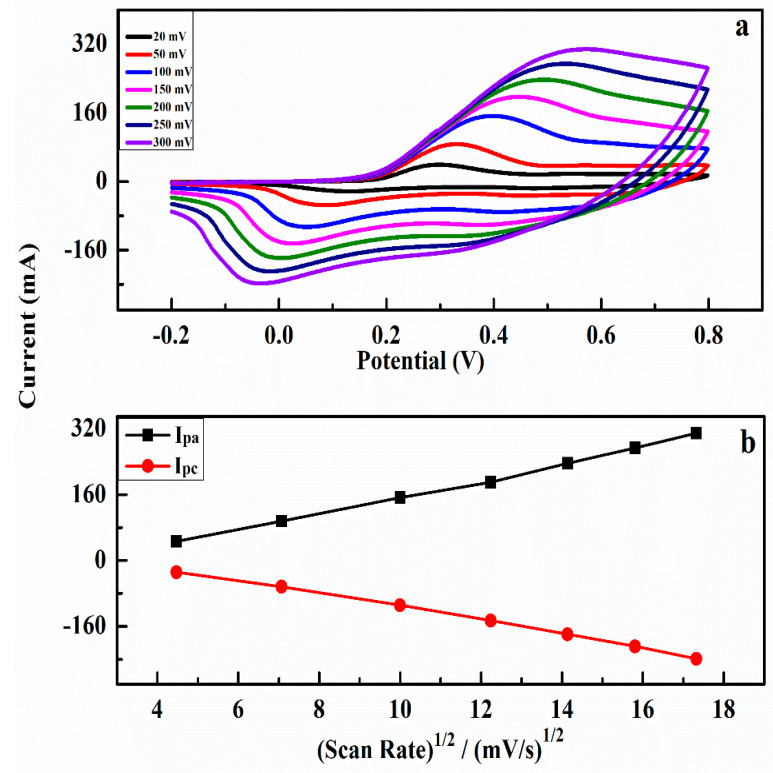
(**a**) CV curves of P-5PA film at various scan rates in 1 M H_2_SO_4_ solution; (**b**) relation of cathodic and anodic peaks of P-5PA against the square route of the scan rate.

The capacitance behavior of P-5PA electrode was further explored by recording the CV curves at a wide range of scan rates (20, 50, 100, 150, 200, 250 and 300 mVs^−1^). The cyclic voltammograms of P-5PA are shown in Figure 5a and the calculated capacitance values are presented in tabulated form (Table 4). It is notable that P-5PA shows excellent electrochemical behavior even in a wide range of scan rates. The CV curves of the P-5PA electrode are quite stable and reflect no aberration even at high scan rates suggesting that the synthesized material is highly stable with respect to charge transfer [28,51]. Furthermore, the specific capacitances of the electrode material decrease with increase in scan rates which is consistent with the above discussed results. The redox processes that take place on the electrode are either kinetically controlled or diffusion controlled. This is proven by plotting the square root of the scan rage against the peak currents of the anodic and cathodic process of the redox transition (Figure 5b). The linear relationship implies that the redox reaction is diffusion controlled within the range of 20–300 mVs^−1^, suggesting the good rate capability of the electrode [57].

The behavior of material at different scan rates is also important for observation of its high-power characteristics which is also an important property of a material for its successful application in different technologies involving SCs. The increase in current densities with scanning rates illustrated the excellent stability, reversibility, and quick response to redox processes [55]. These outcomes publicized a high-power delivery of the PANI materials as electroactive electrode materials in SCs applications. The enhancement in the electrochemical properties originates from the highly percolative electron conduction pathway and is expected due to our ultra-fast synthetic route by using sodium phytate as novel dopant.

#### 3.3.2. Galvanostatic Charge/Discharge (GCD)

**Figure 6:**
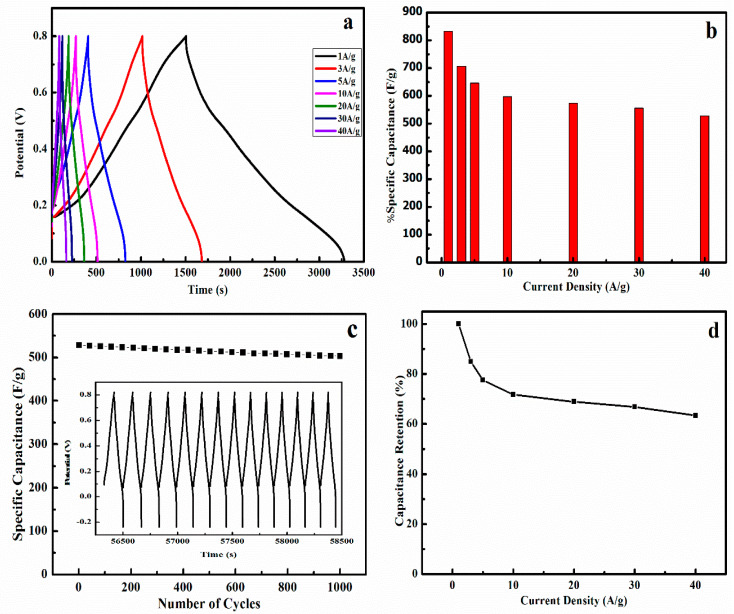
(**a**) GCD curves of P-5PA at current densities of 1, 3. 5, 10, 15, 20, 30 and 40 Ag^−1^ (**b**) Histogram showing specific capacitance values of P-5PA at various current densities; (**c**) cycle stability of P-5PA over 1000 GCD cycles at 40 Ag^−1^ while inset reflects few GCD cycles at 40 Ag^−1^ current density; (**d**) percent capacitance retention of P-5PA against various current densities.

Due to comparatively high specific capacitance both at low and high scan rates, the possibility of the P-5PA electrode as the potential application for capacitors is higher than others. Therefore, the P-5PA electrode was further subjected to the GCD measurement for the exploration of its capacitive behavior in a three-electrode system. Figure 6a illustrates GCD curves of P-5PA measured at a wide range of current densities (1 Ag^−1^ to 40 Ag^−1^). All the GCD curves of the PANI salt showed nearly symmetric and equilateral triangular shapes, which are characteristic of an excellent capacitance and high reversibility of PANI salts during the charging/discharging process [58]. The deviation of discharge curves from a linear shape is an evidence of pseudo capacitance behavior due to the quick response of redox processes [45,59]. The specific capacitances from the GCD curves were assessed by using the following equation [25] and are illustrated in the Table 5 and Figure 6b.

The results indicate that at a low current density of 1 Ag^−1^, the P-5PA electrode shows a maximum value 832.5 ±1.40 F g^−1^ and this remains 528.0 ±1.36 F g^−1^ when the current density was increased to 40 Ag^−1^. In this wide range of current densities, this electrode material generated an excellent rate performance of 67.6% of its initial capacitance retention. This shows a profound distinction to formerly stated PANI-based electrodes, where 25–40% losses in capacitance value was observed at a current density of 1-5 Ag^−1^ [60,61]. This high performance could be attributed to the facile ionic and electronic transport emanating from the conductive network of the prepared PANI salt. The specific capacitance of P-5PA electrode is found to be much better than the values as reported for other PANI and PANI based materials for SCs mentioned in (see Supplementary Materials, Table S2) [15,54,62,63,64].

Annotations of the above findings exposed that high capacitance values at low current density of GCD can be attributed due to the sufficient diffusion of counter-anions into and out of PANI during the process of charging/discharging at the lower current density while higher current density contributes to the increase in the ohmic resistance which results in slow diffusion of ions by resisting the electrolyte ion to penetrate the inner surface of active materials [65]. These findings are similar to that observed in CV analysis.

The excellent capacitive performance of the P-5PA electrode can mainly be attributed to the synergistic effect between dopant and PANI backbone in the matrix, which leads to the formation of conductive, rough, and porous nanostructure. The sodium phytate anchored PANI surface can effectively accumulate protons from the electrolyte and also facilitates the transportation of those protons into the inner surface of the electrode by decreasing ion diffusion path even at higher current density. From these finding, we can say that our material has the potential for application in electrochemical SCs.

#### 3.3.3. Cycling Stability

Cycling stability is also a key factor for the operational SCs. SCs based on CPs often experienced limited cyclability because of shrinking and swelling of electroactive polymers during its charging or discharging operation [66]. The cycling performance of P-5PA showed a capacitance retention of 95.3% over 1000 cycles at a high current density of 40 Ag^−1^ (Figure 6c,d). According to the reported literature, this retention is superior to the SCs based on PANI (typically 60∼85% retention for 1000 cycles) [58,67]. The achievement of the high cycling performance for our electrode material could be accredited to the porous and cross-linked nanofibers morphology of sodium phytate doped PANI that can compensate the shrinking and swelling problems of the polymer network during intensive cycling processes.

#### 3.3.4. Electrochemical Impedance Spectroscopy (EIS)

**Figure 7:**
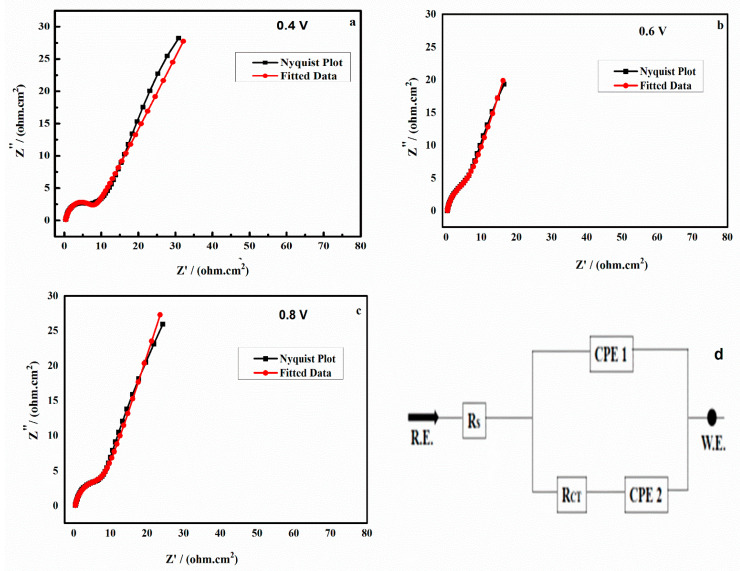
Impedance spectra of P-5PA (**a**) at 0.4 V, (**b**) 0.6 V, (**c**) 0.8V and (**d**) proposed equivalent circuit for P-5PA film.

To evaluate the interfacial charge transfer process at electrode/electrolyte interface, the P-5PA electrode was subjected to EIS analysis and the corresponding Nyquist plots are portrayed in Figure 7 at various potentials within 0.1–10^5^ Hz frequency range. Each spectrum manifests a semicircle that appears in the region of high frequency and an inclined line observed in the region of low frequency. The Surface properties of the electrode are associated with the semicircle as high frequency region involves the electrochemical process at the electrode surface and is responsible for the faradic charge transfer resistance (R_ct_) and double layer capacitance. The inclined line in the low frequency region illustrates the transport and diffusion of the ions due to pseudo-capacitance [45]. The appearance of a vertical line supports the capacitive behavior of the material. If the inclined line is short and close to the *y*-axis, it is attributed to the ideal capacitive behavior [68,69,70].

Nyquist plots were further fitted with a proposed equivalent circuit model illustrated in Figure 7d and parameters are given in Table 6. The proposed equivalent model includes the solution resistance R_s_, the charge transfer resistance R_ct_, a constant-phase element CPE1 to describe the double-layer capacitance at the electrode/electrolyte interface, and a second constant phase element CPE2 to describe the pseudo-capacitance of the PANI material [45]. A decreasing trend of R_ct_ is observed from 7.67 Ω at 0.4 V to 7.44 Ω at 0.6 V followed by an increase in value of R_ct_ to 7.49 Ω at 0.8 V. Similar trend can be found for the pseudo-capacitance described by CPE2, particularly in the an increase of the exponent n up to 0.77 at 0.6 V, which indicates higher capacitive behavior. At 0.8 V this value decreases again to n = 0.71. It is reported that on increasing potential up to a certain limit, an increase in pseudo-capacitive behavior of the electrode material is observed [25,71]. Same observations can be seen in the present work, when we increase the potential from 0.4 to 0.6 V, the plots show dominant pseudo-capacitive behavior but after 0.6 V, the decrease in pseudo-capacitive behavior is observed at 0.8 V. This demonstrates that a synthesized PANI nanofiber by an ultra-fast and green route is a significant and simple way to improve electrochemical properties. The sodium phytate doped PANI nanofibers may have potential applications in energy storage and other electrochemical devices.

## 4. Conclusions

Sodium phytate doped PANI nanofibers were synthesized by a quite simple, scalable, cost-effective, and rapid process (5–10 min), using aniline as monomers, sodium phytate as dopant and APS as an oxidant with a higher conductivity (10 Scm^−1^). SEM results revealed that the optimized sample has fibrous and porous nanostructure networks with distinct connectivity. BET results exhibited a significantly higher surface area value (230.5 m^2^g^−1^) of the optimized sample. The EDX spectra shows that all PANI samples contain C, O, N, Na and P in the polymer back bone, however the Na and P content are higher in the optimized sample P-5PA. FT-IR analysis confirmed the basic functional groups in the synthesized PANI salts. UV-vis analysis revealed three major absorbance peaks which are associated with PANI. The electrochemical results revealed that the optimized sample P-5PA renders impressive capacitive properties. From CV specific capacitance was calculated for a range of scan rates between 20 to 300 mV. At low (20 mV) and high (300 mV) scan rates the specific capacitances of the most optimized sample were 950.0 and 517.5 Fg^−1^_,_ respectively. Similarly, from GCD discharge curves the specific capacitances were calculated for a range of current densities (1 to 40 Ag^−1^). At low (1 Ag^−1^) and high (40 Ag^−1^) current density the capacitance of the optimized sample was 832.5 and 528 Fg^−1^ respectively in three-electrode system. The cyclic stability was checked at a higher current density of 40 Ag^−1^ and shows an excellent retention 95.3% for 1000 cycles. The specific capacitances obtained from both the CV and GCD support each other. Charge transfer resistance (R_ct_) and pseudo-capacitance values for P-5PA were determined from EIS at different applied voltages. Parameters obtained from the proposed equivalent circuit shows that P-5PA at 0.6 V has a small charge transfer resistance (R_ct_) 7.44 ohm and a high pseudocapacitance (n(CPE2) = 0.77). These results demonstrated that the synthesized sodium phytate doped PANI can be effectively used as electrode material in SCs.

## Figures and Tables

**Table 1:** Determined atomic rates of sodium and phosphorous by EDX analysis.

Atomic Ratios	P-0.5PA	P-1PA	P-3PA	P-5PA	P-7PA	P-10PA
Na/%	0.11	0.47	0.69	1.05	0.97	0.89
P/%	0.39	0.55	1.43	1.69	0.98	0.97

**Table 2:** Electrical conductivity (DC) of P-0.5PA to P-10PA at 25 °C.

PANI Sample	P-0.5PA	P-1PA	P-3PA	P-5PA	P-7PA	P-10PA
S/cm	1.37	3.62	5.44	10.0	5.11	3.50

**Table 3:** Anodic peak current (I_pa_), cathodic peak current (I_pc_) and specific capacitance (C_sp_) with standard error of various PANI samples at low (20 mVs^−1^) and high (300 mVs^−1^) scan rate.

**Low Scan Rate (20 mVs^−1^)**			
**Sample Code**	**I_pa_**	**I_pc_**	**C_sp_ (Fg^−1^)**
P-0.5PA	23.30	−22.45	582.5 ± 2.06
P-1PA	32.80	−21.08	819.8 ± 1.81
P-3PA	35.00	−26.81	875.0 ± 1.55
P-5PA	39.00	−30.28	950.0 ± 1.09
P-7PA	33.60	−26.99	840.0 ± 1.95
P-10PA	29.56	−18.97	739.0 ± 2.24
**High Scan Rate (300 mVs^−1^)**			
**Sample Code**	**I_pa_**	**I_pc_**	**C_sp_ (Fg^−1^)**
P-0.5PA	182.34	−124.80	300.5 ± 2.38
P-1PA	225.95	−168.41	378.7 ± 2.10
P-3PA	274.15	−203.60	454.2 ± 1.62
P-5PA	309.34	−238.79	517.5 ± 1.52
P-7PA	252.34	−183.71	424.2 ± 1.96
P-10PA	221.74	−140.10	372.7 ± 2.05

**Table 4:** Anodic peak current (I_pa_), cathodic peak current (I_pc_) and specific capacitance (C_sp_) with standard error of P-5PA at various scan rates.

Scan Rate (mV)	I_pa_	I_pc_	C_sp_ (Fg^−1^)
20	46.2	−28.3	950.0 ± 1.09
50	95.3	−63.6	870.0 ± 1.17
100	152.8	−108.7	766.4 ± 1.12
150	190.3	−146.0	655.3 ± 1.25
200	236.5	−179.3	590.9 ± 1.32
250	273.7	−208.7	548.7 ± 1.47
300	309.1	−238.8	517.5 ± 1.52

**Table 5:** C_sp_ values with standard error of P-5PA against a wide range of current densities.

Current Density (Ag^−1^)	C_sp_(Fg^−1^)
1	832.5 ± 1.40
3	706.5 ± 1.50
5	645.5 ± 1.47
10	597.0 ± 1.59
20	573.0 ± 1.51
30	556.0 ± 1.58
40	528.0 ± 1.36

**Table 6:** Parameters derived from impedance spectra of P-5PA at different voltages.

Voltage (V)	R_ct_ (Ω)	R_s_ (Ω)	CPE1	CPE2	n (CPE2)
0.4	7.674	4.24 × 10^−1^	2.33 × 10^−3^	1.56 × 10^−2^	0.736
0.6	7.439	2.81 × 10^−1^	8.04 × 10^−4^	2.69 × 10^−2^	0.769
0.8	7.490	3.54 × 10^−1^	2.68 × 10^−4^	2.69 × 10^−2^	0.708

## Supplementary Materials

The following are available online at https://www.mdpi.com/2073-4360/12/10/2212/s1, Figure S1: Synthesis of sodium phytate doped PANI. Figure S2: Nitrogen adsorption Curve of PANI-S1 while the inset curve shows pore size distribution and pore volume. Figure S3: Nitrogen adsorption Curve of PANI-S4 while the inset curve shows pore size distribution and pore volume. Figure S4: Nitrogen adsorption Curve of PANI-S6 while the inset curve shows pore size distribution and pore volume. Figures S5, S6 and S7: EDX spectra and mapping of elements for sample S1, S4 and S6, respectively. Table S1: Comparison of selected synthesis methods for polyaniline nanofibers with sodium phytate as dopant. Table S2: Comparison of the Specific Capacitance of PANI and PANI based materials in three-electrode system.
