# Supplementary material for: An Amazingly Simple, Fast and Green Synthesis Route to Polyaniline Nanofibers for Efficient Energy Storage
Source: Polymers (Basel). 2020 Sep 27;12(10):2212. doi: 10.3390/polym12102212 (PMC7600561; doi:10.3390/polym12102212)
Supplement: Supplementary file 1 [file polymers-12-02212-s001.pdf]

polymers

Supporting Information

An Amazingly Simple, Fast and Green Synthesis Route to Polyaniline Nanofibers for Efficient Energy Storage

Sami ur Rahman [1], Philipp Röse [2], Anwar ul Haq Ali Shah [3], Ulrike Krewer [2*] and Salma Bilal [1*]

[1] National Centre of Excellence in Physical Chemistry 1, University of Peshawar, 25120 Peshawar, Pakistan; samiurrahman364@yahoo.com (S.R)

[2] Karlsruhe Institute of Technology (KIT), Institute for Applied Materials – Materials for Electrical and Electronic Engineering (IAM), 76131 Karlsruhe, Germany; ulrike.krewer@kit.edu (U.K)

[3] Institute of Chemical Science, University of Peshawar, 25120 Peshawar, Pakistan; anwarulhaqalishah@uop.edu.pk (A.A.S)

* Correspondence: salmabilal@uop.edu.pk; Tel.: 0049-531-39163651 or 0092-919216766 (S.B.); ulrike.krewer@kit.edu; Tel: +49 721 608-47569 (U.K.)

1. PANI-Synthesis

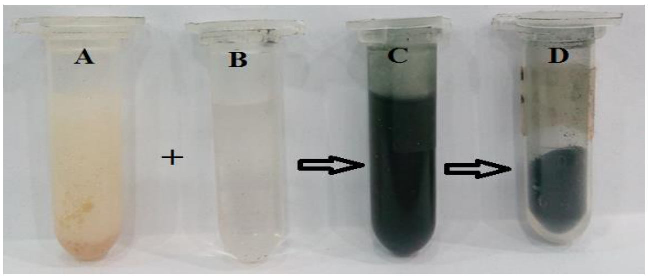

Figure S1: Synthesis of sodium phytate doped PANI.

Table S1. Comparison of selected synthesis methods for polyaniline nanofibers with sodium phytate as dopant.

Method	Solvent	Route	Equipment	Time for final product
Electrochemical[1]	Organic/ Inorganic	Toxic (aqueous or non-aqueous electrolytes such as H_2SO_4, HCl, acetonitrile etc. are employed)	Cell development Electrodes, Potentiostate, electrical energy	More than 50 hours
Chemical[2]	Organic/ Inorganic	Toxic (chloroform, toluene, acetone, acids, etc are largely consumed)	Magnetic stirrer, extensive glass wares, electrical energy	More than 120 hours
Present	Water	Green (no electrolyte or organic solvents required)	Eppendorf's tubes only	5-10 minutes

2. Results of the BET and BJH-Experiments

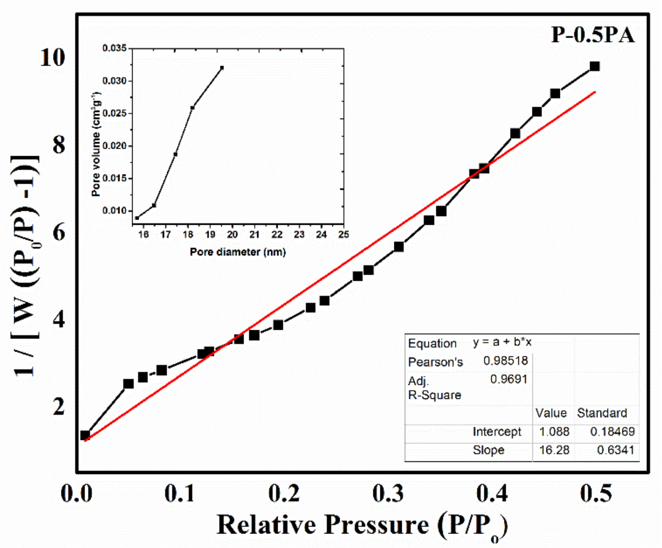

Figure S2. Nitrogen adsorption Curve of PANI-S1 while the inset curve shows pore size distribution and pore volume.

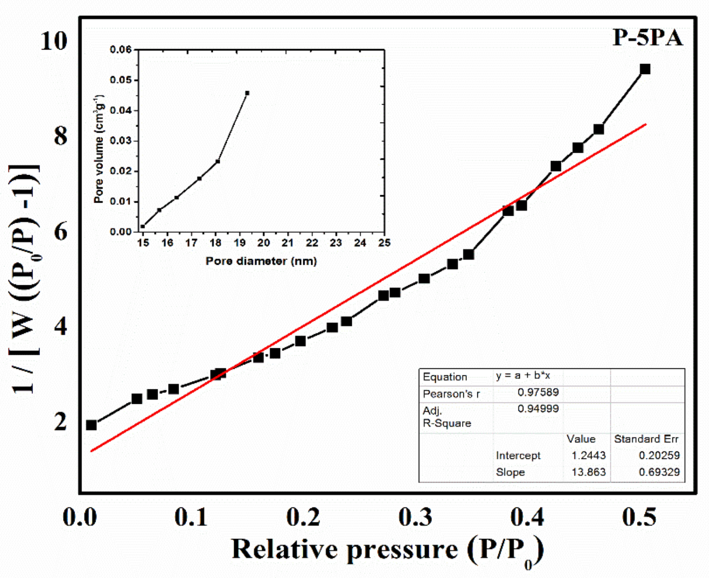

Figure S3. Nitrogen adsorption Curve of PANI-S4 while the inset curve shows pore size distribution and pore volume.

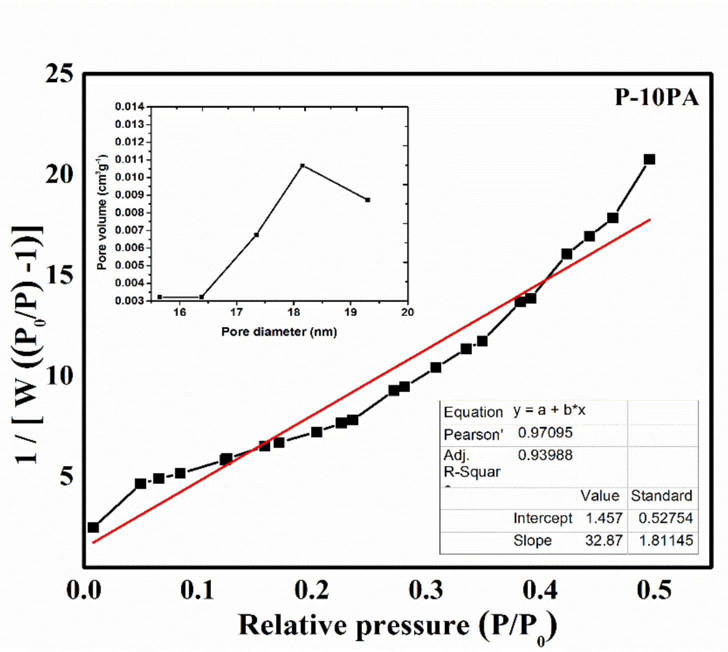

Figure S4. Nitrogen adsorption Curve of PANI-S6 while the inset curve shows pore size distribution and pore volume.

3. Results of the EDX-Mapping

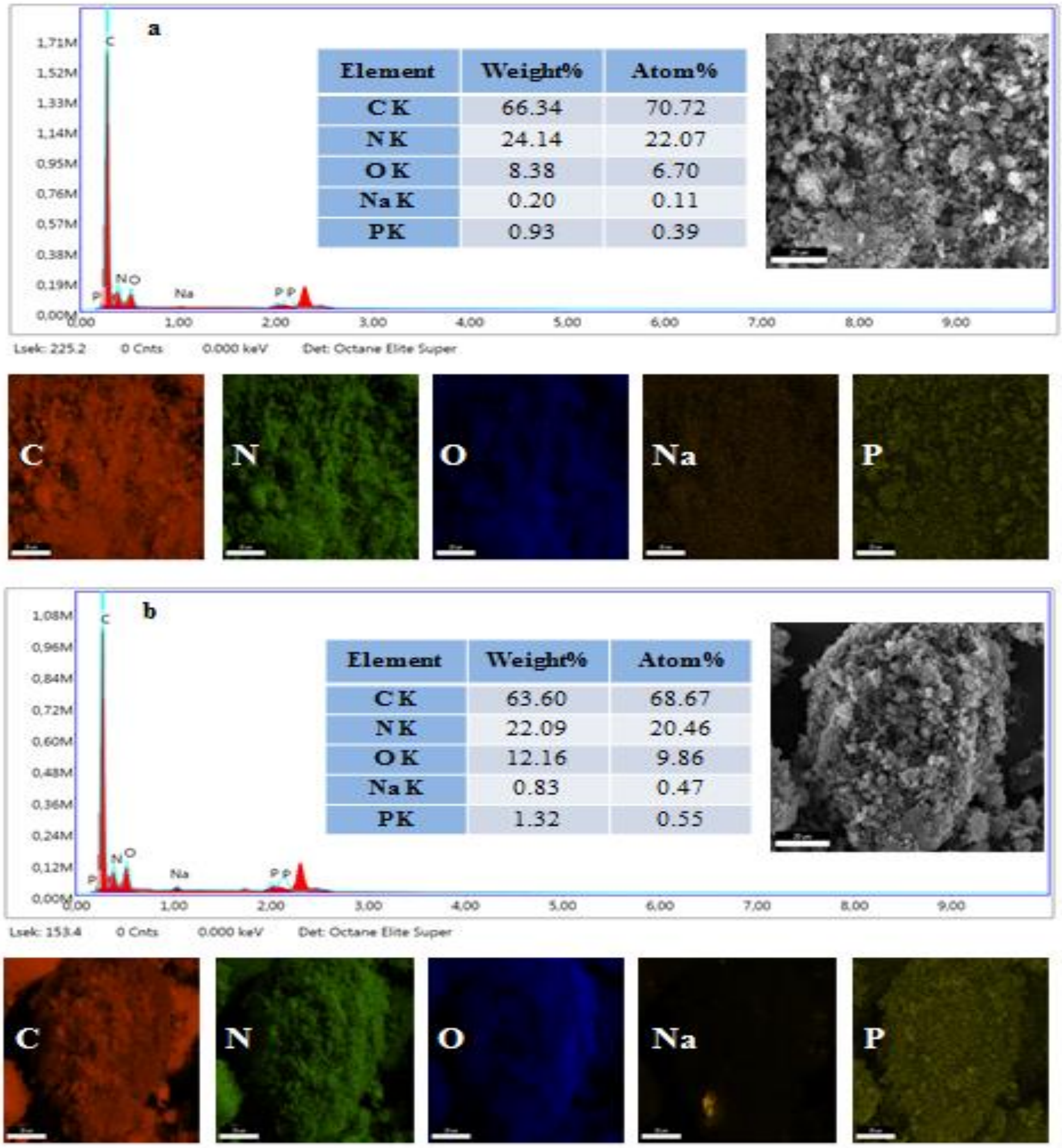

Element	Weight%	Atom%
C K	66.34	70.72
N K	24.14	22.07
O K	8.38	6.70
Na K	0.20	0.11
P K	0.93	0.39

Element	Weight%	Atom%
C K	63.60	68.67
N K	22.09	20.46
O K	12.16	9.86
Na K	0.83	0.47
P K	1.32	0.55

Figure S5: EDX and EDX-mapping of a) P-0.5PA and b) P-1PA

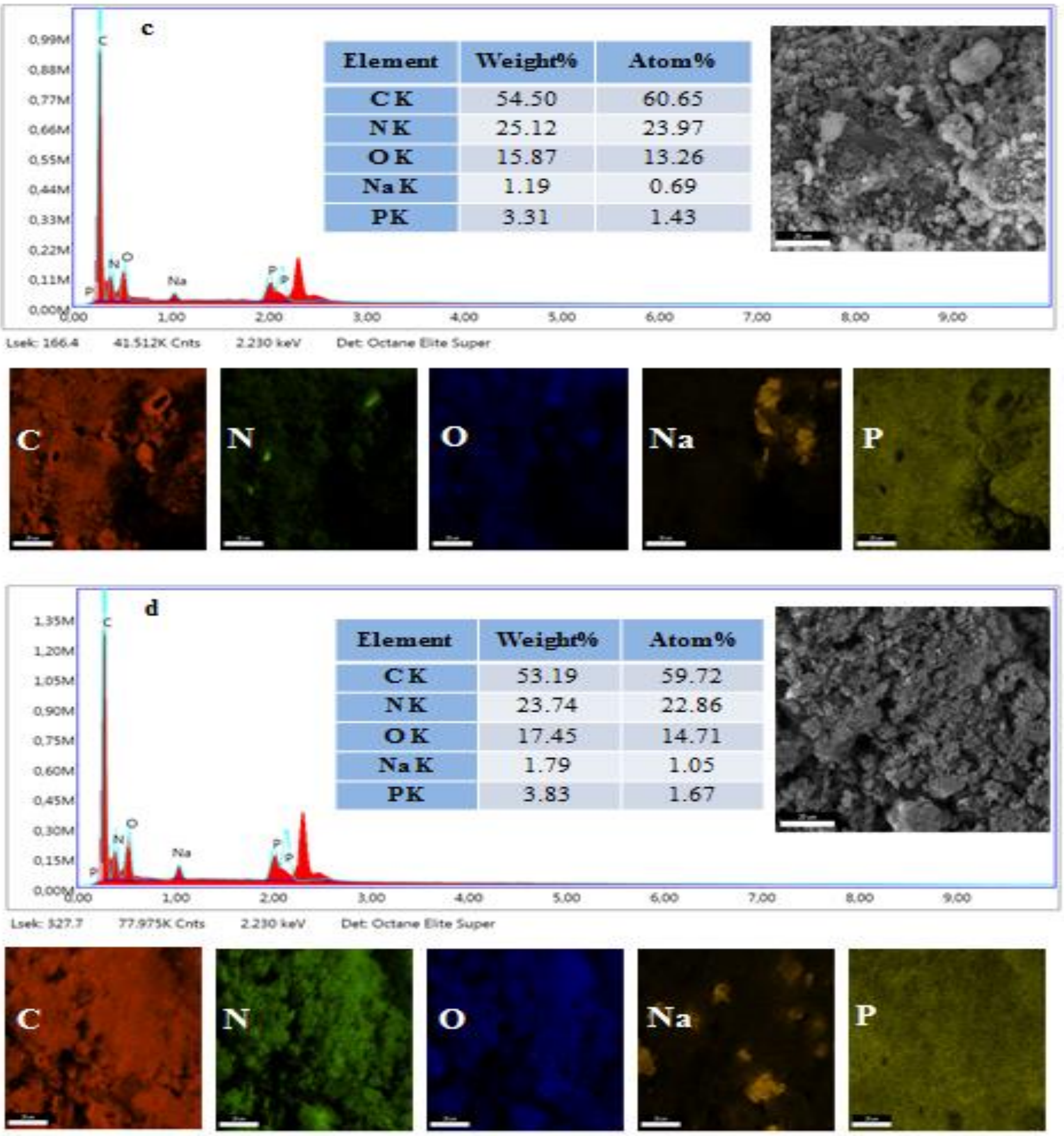

Figure S6: EDX and EDX-mapping of c) P-3PA and b) P-5PA

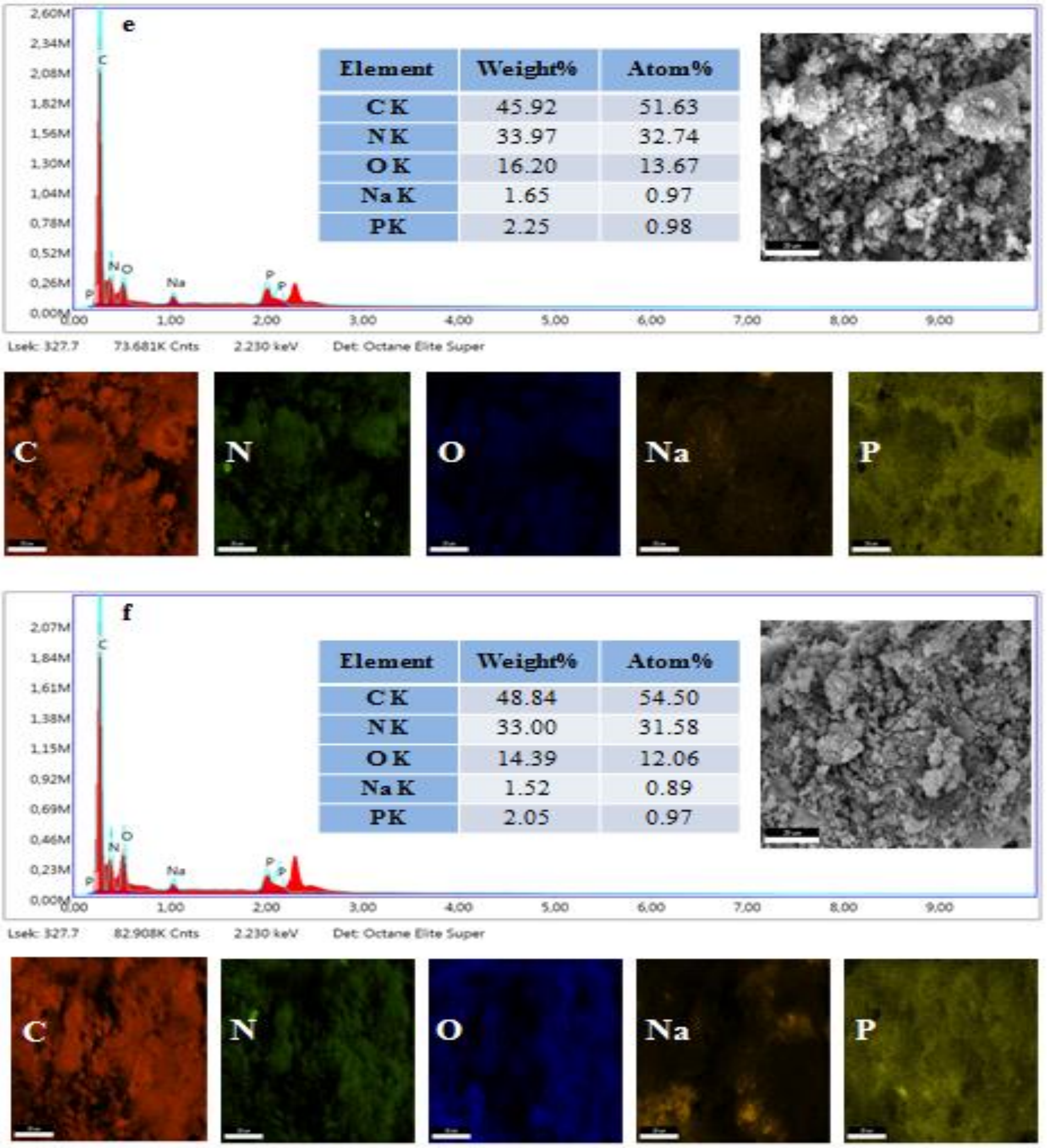

(e)

Element	Weight%	Atom%
C K	45.92	51.63
N K	33.97	32.74
O K	16.20	13.67
Na K	1.65	0.97
P K	2.25	0.98

(f)

Element	Weight%	Atom%
C K	48.84	54.50
N K	33.00	31.58
O K	14.39	12.06
Na K	1.52	0.89
P K	2.05	0.97

Figure S7: EDX and EDX-mapping of e) P-7PA and f) P-10PA

Table S2: Comparison of the Specific Capacitance of PANI and PANI based materials in three electrode system.

Materials	Current Density	Capacitance	Electrolyte	Year	Reference
PANI	$0.5Ag^{-1}$	$712\ Fg^{-1}$	1 M H_2SO_4	2019	[15]
Cl-PANI NFs	$30\ Ag^{-1}$	$105\ Fg^{-1}$	0.1 M HCl	2019	[54]
PhA-PANI NFs	$30\ Ag^{-1}$	$227\ Fg^{-1}$	0.1 M HCl	2019	[54]
Carbon coated PANI	$1\ Ag^{-1}$	$783\ Fg^{-1}$	1 M H_2SO_4	2019	[62]
Honeycomb like PANI	$1\ Ag^{-1}$	$480\ Fg^{-1}$	1 M H_2SO_4		
PANI nanocomposite	$1\ Ag^{-1}$	$626\ Fg^{-1}$	0.5 M H_2SO_4		
PANI nanocomposite	$10\ Ag^{-1}$	$475\ Fg^{-1}$	0.5 M H_2SO_4		
Sodium phytate doped PANI	$1\ Ag^{-1}$	$832.5\ Fg^{-1}$			

© 2020 by the authors. Submitted for possible open access publication under the terms and conditions of the Creative Commons Attribution (CC BY) license (http://creativecommons.org/licenses/by/4.0/).
